# Increased expression of miR-222 is associated with poor prognosis in bladder cancer

**DOI:** 10.1186/1477-7819-12-241

**Published:** 2014-07-31

**Authors:** Dong-qing Zhang, Chang-kuo Zhou, Xue-wen Jiang, Jun Chen, Ben-kang Shi

**Affiliations:** 1 Department of Urology, Qilu Hospital of Shandong University, Jinan 250012, People's Republic of China

**Keywords:** Bladder cancer, microRNA-222, Prognosis

## Abstract

**Background:**

MicroRNA-222 (miR-222) has been shown to play a potential oncogenic role in bladder cancer. The aim of this study was to evaluate the expression of miR-222 in bladder cancer and its potential relevance to clinicopathological characteristics and patient survival.

**Methods:**

Surgical specimens of cancer tissue and adjacent normal tissue were obtained from 97 patients with bladder cancer. The relative expression levels of miR-222 in the cancer and the normal adjacent tissue were measured by quantitative reverse-transcriptase PCR. We analyzed their correlation with clinicopathological parameters and prognostic value.

**Results:**

The expression level of miR-222 was significantly higher in tumor tissues than in corresponding non-cancerous tissues (5.46 ± 1.45 versus 1.92 ± 0.65, *P* < 0.0001), and a high expression of miR-222 was found to be significantly associated with tumor grade (*P* = 0.003) and tumor stage (*P* = 0.005). The miR-222 expression level was classified as high or low in relation to the median value (cutoff value = 5.15). Kaplan-Meier analysis showed that patients with higher levels of miR-222 had significantly poorer survival than those with lower expression of this miRNA in patients, with a 5-year overall survival of 29.53% and 52.75%, respectively (*P* = 0.0034). In the multivariate Cox proportional hazards analysis, which included miR-222 level, tumor grade, tumor stage, and tumor number, high miR-222 expression was independently associated with poor survival (*P* < 0.001; hazard ratio 6.17; 95% CI 2.33 to 10.39).

**Conclusion:**

miR-222 overexpression is involved in the poor prognosis of bladder cancer and can be used as a biomarker for selection of cases requiring special attention.

## Background

Bladder cancer is the thirteenth leading cause of cancer death worldwide [1]. The age-adjusted incidence rate was 20.8 per 100,000 men and women per year, and the age-adjusted death rate was 4.4 per 100,000 men and women per year from 2006 to 2010. The incidence of bladder cancer increases with age, peaking between 50 and 70 years, and the disease is approximately three times more common in males than in females [2].

miRNAs have been identified as signatures associated with diagnosis, staging, progression, prognosis, and response to treatment in human cancer [3-5]. Mature miRNAs are small (20 to 21 nucleotides in length) endogenous non-coding RNAs that regulate the expression of target genes at the post-transcription level through degradation of transcripts and inhibition of translation by mainly binding to the 3’-UTR of target mRNA [6]. The functions of miRNA have been shown to be involved in various critically important physiological processes such as cell proliferation, cell division, cell differentiation, cell apoptosis, tumorigenesis, hematopoiesis, and patterning of the nervous system [7,8].

microRNA-222 (miR-222) belongs to the miR-221/222 family, which was encoded in tandem on chromosome X. Recently, accumulating evidence has demonstrated that miR-222 plays a crucial role in cancer cell proliferation [9], and overexpression of miR-222 has been found in several types of cancers such as breast cancer [10], colorectal carcinoma [11], glioblastoma [12], ovarian cancer [13], prostate cancer [14], and pancreatic cancer [15]. However, the expression of miR-222 in bladder cancer and its prognostic values still remain unclear. The aim of the present study is to evaluate the clinical significance of miR-222. The expression level of the miR-222 was measured in both adjacent normal tissue and cancerous tissue. Furthermore, the correlation between the expression level of miR-222 and clinicopathological characters was analyzed. In addition, the influence of miR-222 on the prognosis of bladder cancer patients was estimated.

## Methods

### Study cohort and samples

This study was approved by the Research Ethics Committee of Qilu Hospital. Written informed consent was obtained from all of the patients. All specimens were handled and made anonymous according to the ethical and legal standards. Clinicopathological data including age, gender, number of tumors, pathological stage, and tumor grade were collected. Patient characteristics are shown in Table 1. None of the patients recruited in this study had undergone preoperative chemotherapy or radiotherapy. For each case, the diagnosis and the histologic grade were confirmed by two pathologists. The duration of follow-up was calculated from the date of surgery to death or last follow-up, and patients were excluded if they had incomplete medical records or inadequate follow-up. For quantitative reverse-transciptase polymerase chain reaction (qRT-PCR), 97 pairs of fresh bladder cancer and matched adjacent normal tissue specimens were collected from patients who underwent surgery between May 2008 and August 2012 in Qilu Hospital. The fresh tissue specimens were collected and immediately placed in liquid nitrogen and then stored at -80°C until the isolation of RNA.

**Table 1:** Association between microRNA-222 expression and different clinicopathological features of bladder cancer

**Variable**	**n**	**Relative microRNA-222 level**	***P *** **value**
Gender			
Female	46	5.33 ± 1.22	
Male	51	5.56 ± 1.53	0.65
Age (years)			
<60	59	5.01 ± 1.34	
≥60	38	5.79 ± 1.49	0.17
Number of tumors			
Single	73	4.97 ± 1.23	
Multiple	24	5.88 ± 1.92	0.09
Stage			
Ta-T1	82	4.19 ± 1.32	
≥T2	15	8.11 ± 1.46	0.005
Grade			
G1/2	39	3.42 ± 1.27	
G3	58	7.91 ± 1.57	0.003

### RNA isolation and qRT-PCR

Total RNA was isolated from frozen specimens by homogenizing tissue in Trizol reagent (Invitrogen, Carlsbad, CA, USA) according to the manufacturer’s instructions. The purity and concentration of RNA were determined using a NanoDrop 1000 spectrophotometer (Thermo Scientific, Wilmington, DE, USA). The differentially expressed amount of the miR-222 was validated in triplicate by qRT-PCR. Briefly, 2 μg RNA was added to the RT reaction; the cDNA then served as the template for amplification of PCR with sequence-specific primers (Sangon Biotech, Shanghai, China) using the SYBR PrimeScript miRNA RT-PCR kit (Takara Biotechnology Co. Ltd, Dalian, China) on the 7500 Real-Time PCR systems (Applied Biosystems, Carlsbad, CA, USA). The PCR cycling profile was denatured at 95°C for 30 seconds, followed by 40 cycles of annealing at 95°C for 5 seconds, and extension at 60°C for 34 seconds. Small nucleolar RNA U6 was used as an internal standard for normalization. The cycle threshold (C_T_) value was calculated. The 2^-ΔCT^ (ΔC_T_ = C_TmiR222_-C_TU6 RNA_) method was used to quantify the relative amount of miR-222. Real-time PCR primers used were: miR-222 - (forward) 5'-CGCAGCTACATCTGGCTACTG-3', (reverse) 5'-GTGCAGGGTCCGAGGT-3'; U6 - (forward) 5'-GCGCGTCGTGAAGCGTTC-3’, (reverse) 5’-GTGCAGGGTCCGAGGT-3'.

### Statistical analysis

The comparison of the expression level of miR-222 between bladder cancer tissue and adjacent normal tissue was performed using the two-sample Student’s *t* test. The correlation between the expression of miR-222 and clinicopathological characters was assessed with the two-sample Student’s *t* test. The overall survival was analyzed by log-rank test, and survival curves were plotted according to Kaplan–Meier. Univariate Cox regression was performed on each clinical covariate to examine its influence on patient survival. Final multivariate models were based on step-wise addition. A Wald statistic of *P* < 0.05 was used as the criterion for inclusion in final multivariate models. All tests were two tailed and results with *P* < 0.05 were considered statistically significant. Statistical analyses were performed using SPSS 13.0 soft-ware (Chicago, IL, USA) and GraphPad Prism 5 (GraphPad Software Inc., CA, USA).

## Results

### Expression of miR-222 in bladder cancer tissues by qRT-PCR

**Figure 1:**
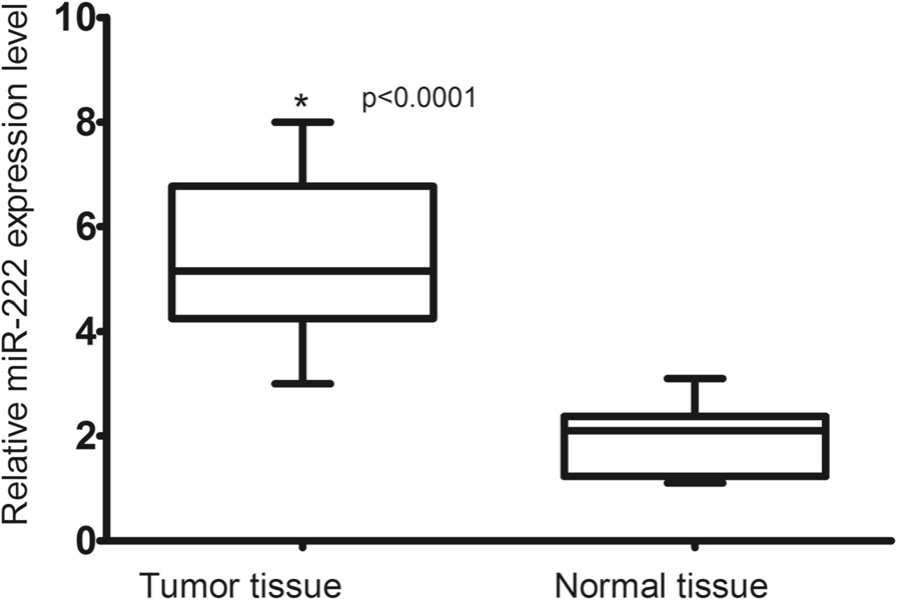
**Comparison of microRNA-222 expression levels between bladder cancer tissue and adjacent normal tissue.** Analysis using the two sample Student’s *t* test showed that the relative expression levels of microRNA-222 (miR-222) in the bladder cancer tissue were significantly higher than those in adjacent normal tissue (*P* < 0.0001).

We examined miR-222 protein expression in 97 pairs of bladder cancer tissues and the corresponding non-cancerous tissues by qRT-PCR. As shown in Figure 1, the expression level of miR222 was significantly higher in tumor tissues than in corresponding non-cancerous tissues (5.46 ± 1.45 versus 1.92 ± 0.65, *P* < 0.0001).

### Correlation of miR-222 expression with clinicopathological characteristics

The relationship between miR-222 expression and different clinicopathological factors is shown in Table 1. Increased miR-222 expression in bladder cancer was found to be significantly associated with tumor grade (Ta-T1 versus ≥ T2: 4.19 ± 1.32 versus 8.11 ± 1.46, *P* = 0.003) and tumor stage (G1/2 versus G3: 3.42 ± 1.27 versus 7.91 ± 1.57, *P* = 0.005). However, no significant correlation was observed between miR-222 expression and other clinicopathologic variables such as age, gender, tumor size, and number of tumors (all *P* > 0.05).

### Relationship between miR-222 expression and survival of bladder cancer patients

**Figure 2:**
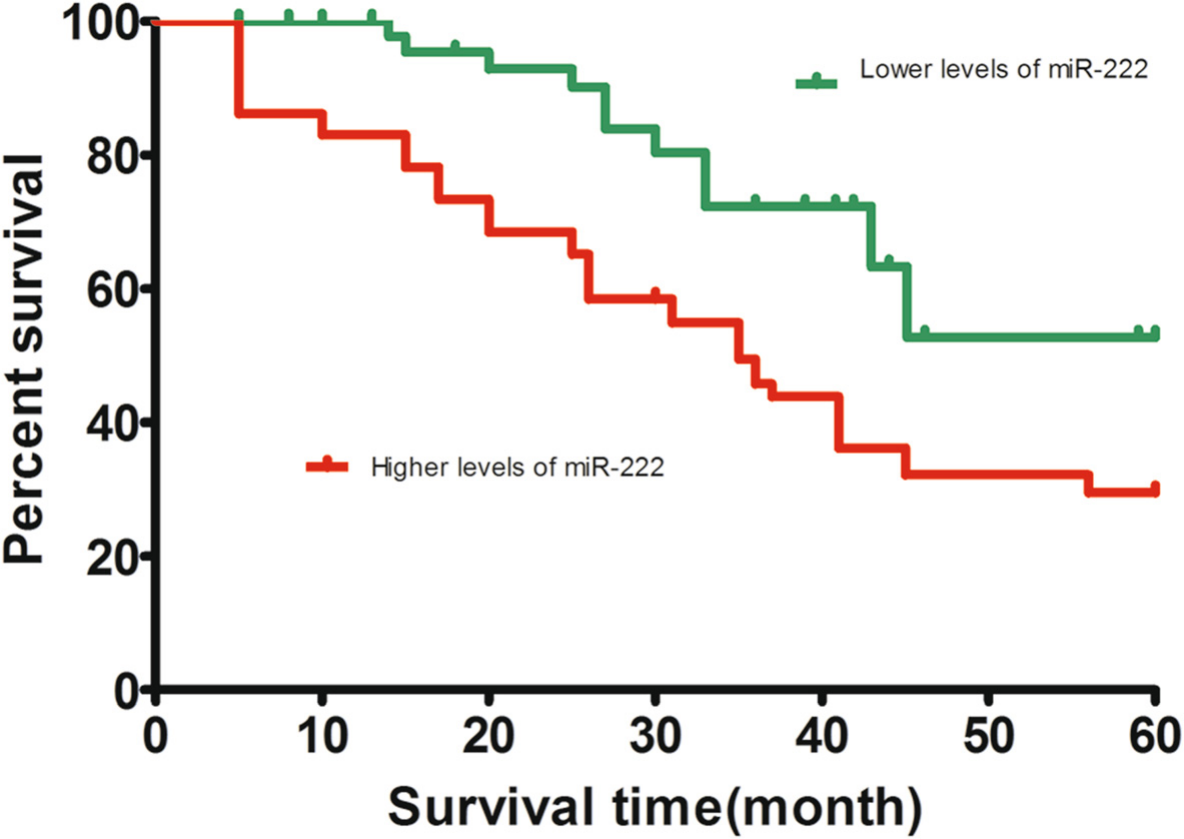
**Kaplan–Meier survival curves in relation to microRNA-222 level in 97 patients with bladder cancer.** The survival rate of patients with high microRNA-222 (miR-222) level was significantly lower than that of patients with low miR-222 level (log-rank test *P* = 0.0034).

**Figure 3:**
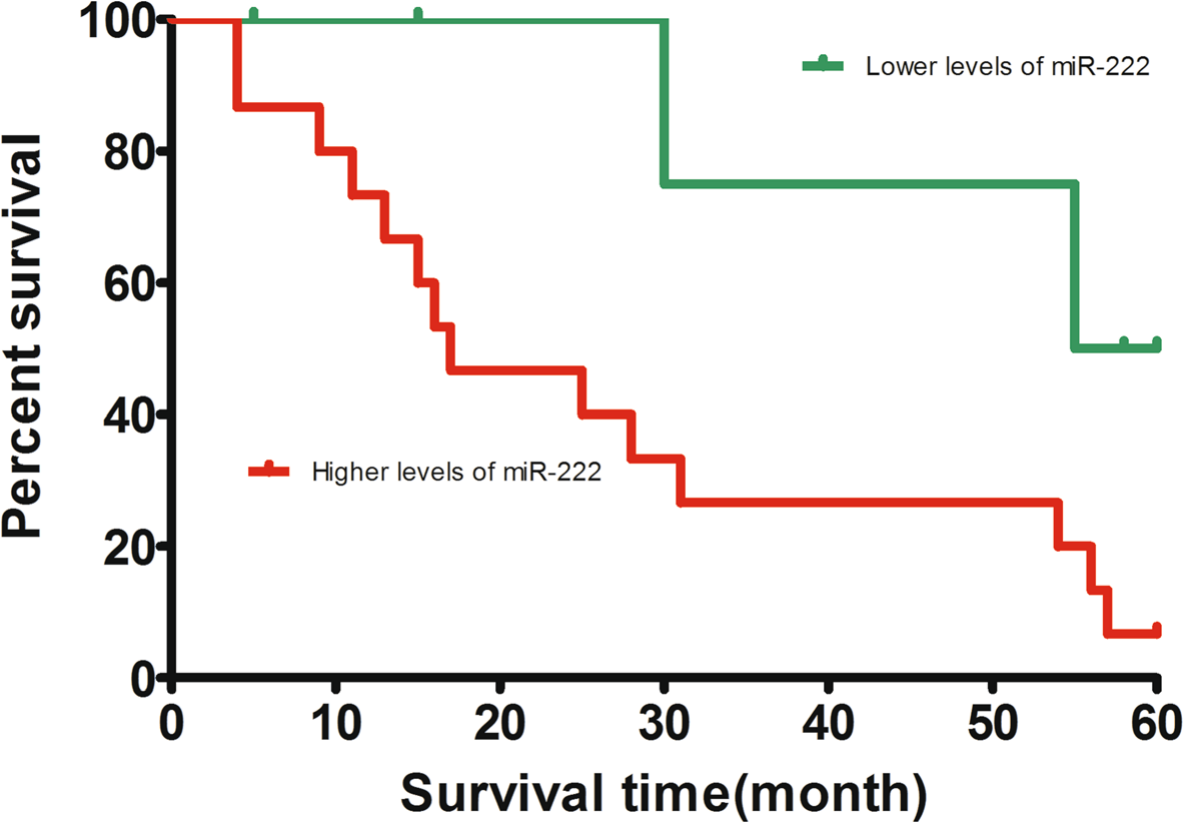
**Kaplan–Meier survival curves in relation to microRNA-222 level in 21 patients with T3G3 bladder cancer.** The survival rate of patients with high microRNA-222 (miR-222) level was significantly lower than that of patients with low miR-222 level (log-rank test *P* = 0.039).

The miR-222 expression level was classified as high or low in relation to the median value (cutoff value = 5.15). To evaluate whether miR-222 expression can predict bladder cancer prognosis, we next performed survival analysis. Kaplan-Meier analysis showed that patients with higher levels of miR-222 had significantly poorer survival than those with lower expression of this miRNA in patients, with a 5-year overall survival of 29.53% and 52.75%, respectively (*P* = 0.0034; Figure 2). Because the survival of G3T3 bladder cancer is much less than G1/2Ta/1/2 bladder cancer (n = 21), we next performed survival analysis for patients diagnosed with G3T3 bladder cancer. Kaplan-Meier analysis showed that patients with higher levels of miR-222 had significantly poorer survival than those with lower expression of this miRNA in patients diagnosed with G3T3 bladder cancer, with a 5-year overall survival of 6.66% and 50.00%, respectively (*P* = 0.039; Figure 3). A Cox proportional hazards analysis was used to further evaluate the potential of miR-222 expression as a prognostic biomarker. Univariate survival analyses indicated that miR-222 expression, tumor stage, and tumor grade were associated with prognosis, while gender, age and tumor number were not associated with prognosis. In the multivariate Cox proportional hazards analysis, which included miR-222 level, tumor grade, tumor stage, and tumor number, high miR-222 expression was independently associated with poor survival (*P* < 0.001; hazard ratio 6.17; 95% CI 2.33 to 10.39; Table 2).

**Table 2:** Multivariate Cox's hazards model analysis for prognostic factors

**Variable**	**Hazard ratio (95% CI)**	***P***
microRNA-222 expression (high versus low)	6.17 (2.33-10.39)	<0.001
Sex distribution (male versus female)	1.29 (0.35-2.34)	0.47
Age (<60 years versus ≥60 years)	1.12 (0.67-1.99)	0.32
Stage (≥T2 versus Ta-T1)	2.98 (1.91-5.69)	0.003
Grade (G3 versus G1/G2)	3.01 (1.78-8.11)	0.002
Number of tumors (multiple versus single)	1.92 (0.92-4.91)	0.11

## Discussion

Identification of miRNA molecular profiles associated with the prognosis of patients with bladder cancer may not only elucidate the underlying biological mechanisms involved in the development or progression of the disease but also provide the opportunity to identify novel targets for bladder cancer therapy. In 2009, Veerla and colleagues found that the expression of miR-222 was obviously upregulated in bladder cancer samples compared with adjacent normal tissues [16]. Recently, Puerta-Gil and colleagues found a significant correlation in quantitative levels of expression between the urinary samples and matching tumors for miR-222 (*P* = 0.008), and qRT-PCR of miR-222 in urine could provide high accuracy for bladder cancer diagnosis [17]. Furthermore, they found that miR-222 expression was significantly correlated with increasing tumor grade (*P* = 0.017), tumor size (*P* = 0.005), presence of carcinoma *in situ* (*P* = 0.035), and clinical outcome endpoints (recurrence, *P* = 0.006; progression, *P* = 0.003; disease-specific survival, *P* = 0.034; and overall survival, *P* = 0.023). However, the expression level of miR-222 and its clinical significance in Asian patients with bladder cancer remains unclear. In the present study, 97 Chinese patients diagnosed with bladder cancer were included. We found that the expression level of miR-222 was significantly higher in tumor tissues than in corresponding non-cancerous tissues. Furthermore, miR-222 expression was proven to be associated with tumor stage and tumor grade, suggesting that miR-222 might be involved in the carcinogenesis and metastasis of bladder cancer. More importantly, we proved that patients with a high expression of miR-222 tended to have shorter survival than patients with lower levels, indicating that a high miR-222 level is a marker of poor prognosis for patients with bladder cancer.

Overexpression of miR-222 has been observed in several types of cancers such as breast cancer [10], colorectal carcinoma [11], glioblastoma [12], ovarian cancer [13], prostate cancer [14], and pancreatic cancer [15], suggesting its important role in tumorigenesis. Lee and colleagues [15] found that the tissue expression levels of miR-222 were significantly upregulated in pancreatic cancer samples compared with those in adjacent normal tissues (*P* = 0.00001). Multivariate analysis with Cox’s proportional hazards model confirmed that the miR-222 high expression level was an independent predictor of poor prognosis for the patients with pancreatic cancer. Furthermore, they found that overexpression of tissue miR-222 strongly related to the expression level of Ki67, which is strictly related to cell proliferation [18]. Hwang and colleagues found that miR-221/222 targets adiponectin receptor 1 to promote the epithelial-to-mesenchymal transition in breast cancer [10]. Sun and colleagues [13] found that miR-222 expression was upregulated in ovarian cancer. Furthermore, their results also provided functional evidence concerning the possible role of miR-222 in ovarian cancer, as it was demonstrated that miR-222 upregulation is able to induce an enhancement of ovarian cancer cell proliferation potential, possibly by downregulating its target, P27^Kip1^. A bioinformatic analysis showed that the 3'-UTR of the P27^Kip1^ mRNA contained the highly conserved putative miR-222 binding site [13]. Quintavalle and colleagues found that miR-222 regulates glioma tumorigenesis at least in part through the control of PTPμ protein expression [12].

In this study, we confirmed the clinical importance of miR-222 expression by showing its association with unfavorable clinicopathological features and poor survival. However, the precise molecular mechanisms behind the altered expression of miR-222 in bladder cancer are unclear. Recently, Calderaro and colleagues found that miR-222 was involved in the PI3K/AKT pathway, which is considered to play a major role in bladder carcinogenesis [19]. Additional studies to investigate the molecular mechanisms of both the cause and the effects of altered expression of miR-222 in the development and/or progression of bladder cancer are essential.

## Conclusion

In conclusion, our results show that miR-222 was overexpressed in bladder cancer, and its high expression indicated an association with poor prognostic factors and poor survival. To clarify the role of miR-222, as well as its use as a biomarker and in targeting therapy, large worldwide population-based studies with a standard definition of miR-222 expression level are necessary.

## Abbreviations

miR-222: microRNA-222; miRNA: microRNA; qRT-PCR: quantitative reverse transcriptase polymerase chain reaction; UTR: untranslated region.

## Competing interests

The authors declare that they have no competing interests.

## Authors' contributions

DQZ and CKZ designed the study and drafted the manuscript; DQZ, CKZ, XWJ, JC and BKS carried out the expertiments and performed the data analysis. All authors read and approved the final manuscript.
